# Somatic mutations can induce a noninflamed tumour microenvironment via their original gene functions, despite deriving neoantigens

**DOI:** 10.1038/s41416-023-02165-6

**Published:** 2023-02-02

**Authors:** Takamasa Ishino, Shusuke Kawashima, Etsuko Tanji, Toshihide Ueno, Youki Ueda, Sadahisa Ogasawara, Kazuhito Sato, Hiroyuki Mano, Soichiro Ishihara, Naoya Kato, Masahito Kawazu, Yosuke Togashi

**Affiliations:** 1 Department of Tumor Microenvironment, Okayama University, Graduate School of Medicine Dentistry and Pharmaceutical Sciences, 2-5-1 Shikata-cho, Kita-ku, Okayama, 700-8558 Japan; 2 Division of Cell Therapy, Chiba Cancer Center Research Institute, 666-2, Nitona-cho, Chuo-ku, Chiba 260-8717 Japan; 3 Department of Gastroenterology, Graduate School of Medicine, Chiba University, 1‑8‑1 Inohana, Chuo‑ku, Chiba 260‑8670 Japan; 4 Department of Dermatology, Graduate School of Medicine, Chiba University, 1‑8‑1 Inohana, Chuo‑ku, Chiba 260‑8670 Japan; 5 Division of Cellular Signaling, National Cancer Center Research Institute; 5-1-1 Tsukiji, Chuo-ku, Tokyo 104-0045 Japan; 6 Department of Surgical Oncology, Graduate School of Medicine, The University of Tokyo; 7-3-1 Hongo, Bunkyo-ku, Tokyo 113-8655 Japan

**Keywords:** Tumour immunology, Cancer immunotherapy, Tumour biomarkers, Immunotherapy, Colorectal cancer

## Abstract

**Background:**

Identifying biomarkers to predict immune checkpoint inhibitor (ICI) efficacy is warranted. Considering that somatic mutation-derived neoantigens induce strong immune responses, patients with a high tumour mutational burden reportedly tend to respond to ICIs. However, there are several conflicting data. Therefore, we focused on the original function of neoantigenic mutations and their impact on the tumour microenvironment (TME).

**Methods:**

We evaluated 88 high-frequency microsatellite instability (MSI-H) colorectal cancers and analysed the function of the identified neoantigenic mutations and their influence on programmed cell death 1 (PD-1) blockade efficacy. The results were validated using The Cancer Genome Atlas (TCGA) datasets.

**Results:**

We identified frameshift mutations in *RNF43* as a common neoantigenic gene mutation in MSI-H tumours. However, loss-of-function *RNF43* mutations induced noninflamed TME by activating the WNT/β-catenin signalling pathway. In addition, loss of *RNF43* function induced resistance to PD-1 blockade even in neoantigen-rich tumours. TCGA dataset analyses demonstrated that passenger rather than driver gene mutations were related to the inflamed TME in diverse cancer types.

**Conclusions:**

We propose a novel concept of “paradoxical neoantigenic mutations” that can induce noninflamed TME through their original gene functions, despite deriving neoantigens, suggesting the significance of qualities as well as quantities in neoantigenic mutations.

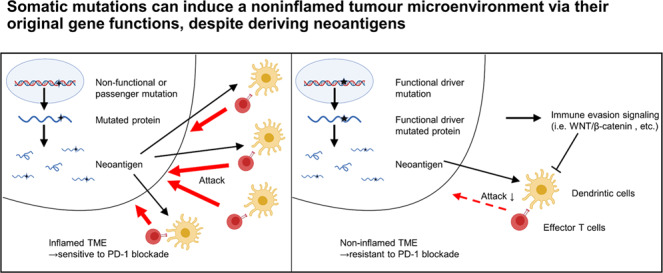

## Background

Cancer develops through the acquisition of immune escape mechanisms [1], including the use of suppressive immune checkpoint molecules such as programmed cell death-1 (PD-1) and PD-1 ligand 1 (PD-L1). Antitumor immune responses can be restored by blocking these immunosuppressive pathways [2]. Cancer immunotherapy with immune checkpoint inhibitors (ICIs), including monoclonal antibodies (mAbs) against cytotoxic T lymphocyte-associated protein 4 (CTLA-4) and PD-1/PD-L1, rescues dysfunctional cytotoxic effector CD8^+^ T cells, leading to tumour regression [3, 4]. ICIs have been demonstrated to be effective against diverse cancer types, including gastrointestinal cancers, leading to a paradigm shift in cancer treatment [5–7]. However, more than half of patients do not respond to ICIs, and identifying biomarkers to predict clinical benefits is imperative.

Somatic mutation-derived neoantigens, which can be recognised as non-self-antigens, reportedly induce strong immune responses similar to those of foreign antigens [8, 9]. Thus, neoantigens are presumed to induce an inflamed tumour microenvironment (TME), which is substantial for the ICI response, and the number of neoantigens is reportedly correlated with the inflamed TME [9–11]. Therefore, tumour mutational burden (TMB) is one of the predictive biomarker candidates for ICIs [10, 12, 13], and ICIs have been approved for treatment of high-frequency microsatellite instability (MSI-H) cancers, which generally have an extremely high TMB [14–16]. However, there are several conflicting data [17–19], and the TMB does not seem to be a sufficient predictor of ICI response. Even in MSI-H cancers, the response rates of PD-1 blockade therapies are only ~50% [20, 21].

Tumours also evade antitumor immunity through “immune editing”, which involves the elimination of highly immunogenic tumour cells or mutation of their own human leukocyte antigen (HLA) molecules to decrease antigen presentation [2, 22–24]. Furthermore, in the process of accumulating somatic mutations, certain cancer signalling pathways promote immune evasion [25–30]. Therefore, quantitative assessment of somatic mutations seems to be insufficient for predicting ICI response, and it can be essential to elucidate the effects of mutated gene functions on the TME. In this study, we focused on the functions of neoantigenic driver gene mutations in MSI-H colorectal cancers. In our MSI-H cohort [31], neoantigens derived from mutations in *RNF43* were commonly observed, as previously reported [32, 33]. However, tumours with *RNF43* mutations did not always have an inflamed TME. We demonstrated that some forms of mutated *RNF43* could suppress antitumor immunity and induce resistance to PD-1 blockade, even when neoantigens were present, by activating the WNT/β-catenin signalling pathway [29, 30, 34, 35]. These results were validated using The Cancer Genome Atlas (TCGA) datasets. We propose a novel concept of “paradoxical neoantigenic mutations” that can induce the noninflamed TME due to their original gene functions, despite deriving neoantigens. When evaluating neoantigens, it is essential to consider the functions of the neoantigenic gene mutations in addition to the number of neoantigens, as the functions of mutated molecules, such as mutated *RNF43*, can affect antitumor immunity.

## Methods

### Study design

Eighty-eight patients with MSI-H colorectal cancer who underwent surgery were enrolled in this study (Table S1) [31]. Written informed consent was obtained prior to participation. The clinical information of patients was obtained from their medical records.

### Whole-exon sequencing

Exome sequencing data were obtained from a publicly available dataset [31]. Genomic DNA was isolated from each sample and subjected to exonic fragment enrichment using a SureSelect Human All Exon Kit v5 (Agilent Technologies, Santa Clara, CA). Massively parallel sequencing of isolated fragments was conducted with a HiSeq2500 (Illumina, San Diego, CA) using the paired-end option. Paired-end whole-exon sequencing (WES) reads were independently aligned to a human reference genome (hg38) using BWA [36], Bowtie2 (http://bowtie-bio.sourceforge.net/bowtie2/index.shtml), and NovoAlign (http://www.novocraft.com/products/novoalign/). Somatic mutations were called using MuTect (http://www.broadinstitute.org/cancer/cga/mutect), SomaticIndelDetector (http://www.broadinstitute.org/cancer/cga/node/87), and VarScan (http://varscan.sourceforge.net). Mutations were discarded if (i) the read depth was <20 or the variant allele frequency was <0.1, (ii) they were supported by only one genome strand, or (iii) they were present in normal human genomes in either the 1000 Genomes Project dataset (http://www.internationalgenome.org/) or our in-house database. Gene mutations were annotated with SnpEff (http://snpeff.sourceforge.net).

### RNA-sequencing

Transcriptome sequencing data were obtained through a previous study; these data have already been reported and are publicly available [31]. Total RNA was extracted using RNA Bee reagent (Tel-Test Inc., Friendswood, TX) and treated with DNase I (Qiagen, Venlo, NL) using a Rneasy Mini Kit (Qiagen). RNA integrity was evaluated using either a Bioanalyzer (Agilent Technologies) or TapeStation (Agilent Technologies). RNA with a high RNA integrity number underwent RNA-seq using an NEBNext Ultra Directional RNA Library Prep Kit (New England BioLabs, Ipswich, MA), with complementary DNA (cDNA) prepared from polyA-selected RNA. RNA with a low RNA integrity number was subjected to RNA-seq using a TruSeq RNA Access Library Prep Kit (Illumina), with cDNA generated using random primers. Prepared RNA-seq libraries underwent next-generation sequencing of 120 bp from both ends (paired-end reads). The expression level of each gene was computed using DESeq2 (http://bioconductor.org/packages/release/bioc/html/DESeq2.html) with VST transformation, and gene fusions were detected using a deFuse pipeline (https://bitbucket.org/dranew/defuse) and STAR (https://github.com/alexdobin/STAR).

### Long-read sequencing

We determined the precise sequences of ~3500 bp regions between the 5′ and 3′ untranslated region (UTR) of HLA-ABC gene using single-molecule real-time sequencing conducted on a Sequel platform (Pacific Biosciences, Menlo Park, CA). The genotypes of HLA-A, HLA-B, and HLA-C genes were determined by comparing the obtained sequences with reference sequences and were subsequently validated using NGSengine software.

### Neoantigen prediction

Neoantigens were predicted as reported in a previous study [24]. We assumed that the genomic DNA sequences carrying somatic nonsynonymous mutations that were also detected in transcriptome sequencing reads produced abnormal peptides. The affinities of abnormal peptides (nine amino acids long) for MHC class I molecules in individual tumours were predicted using NetMHCpan (Ver. 4.0) [37]. Abnormal peptides that were presumed to result from frameshift (fs) mutations were also included in our cohort analysis. The peptides in the top two percentiles for affinity were defined as neoantigens.

### Immunohistochemistry

Formalin-fixed paraffin-embedded (FFPE) specimens from human clinical samples were cut into 4 µm sections and mounted on glass slides. Antigen retrieval was performed in citrate buffer (pH 6) at 121 °C for 20 min. CD8^+^ cell infiltration within the tumour gland was observed and blindly scored by an experienced pathologist according to the following criteria: 0 (0–5% CD8 staining); 1 (5–10% CD8 staining); 2 (10–15% CD8 staining); and 3 (>15% CD8 staining). Simultaneously, CD8^+^ cells were quantified using an automated imaging system (Vectra 3.0; Perkin-Elmer, Waltham, MA, USA) equipped with the analytical software InForm 2.2 (Perkin-Elmer).

Frozen tissue specimens from mice were cut into 6 µm sections and mounted on glass slides. Individual slides were incubated overnight at 4 °C with anti-CD11c mAb (D1V9Y; Cell Signalling Technology, Danvers, MA) and anti-CD8 mAb (D4W2Z; Cell Signaling Technology). The slides were then incubated in SignalStain^®^ Boost IHC Detection Reagent (Cell Signaling Technology), and the colour was developed using a Signalstain^®^ DAB Substrate Kit (Cell Signalling Technology). CD11c- and CD8-positive cells were counted; three fields (×400) comprising tumour cells were randomly selected and counted for each slide. The mean of the three area counts for each tumour was used for statistical analysis.

### Gene set enrichment analysis

Enriched pathways were determined using the GSEA tool available from the Broad Institute website (http://software.broadinstitute.org/gsea/index.jsp) [38].

### Cell lines and reagents

The HEK293T (human embryonic kidney cell), CT26 (murine colon cancer), RENCA (murine renal cell carcinoma), and A20 (murine lymphoma) cell lines were purchased from ATCC (Manassas, VA). The HEK293T and CT26 cell lines were maintained in DMEM (FUJIFILM Wako Pure Chemical Corporation, Osaka, Japan) supplemented with 10% foetal calf serum (FCS; Cytiva, Tokyo, Japan). The RENCA and A20 cell lines were maintained in RPMI medium (FUJIFILM Wako Pure Chemical Corporation) supplemented with 10% FCS. All tumour cells were confirmed to be *Mycoplasma* (−) using a PCR Mycoplasma Detection Kit (TaKaRa, Shiga, Japan) according to the manufacturer’s instructions before use. Recombinant human Wnt3a protein was obtained from R&D (Minneapolis, MN). Recombinant murine Wnt3a protein was purchased from PeproTech (Cranbury, NJ). Rat anti-mouse PD-1 mAb (RMP1-14) and control rat IgG2a mAb (RTK2758) were obtained from BioLegend (San Diego, CA).

### Constructs, virus production, and transfection

The pGreenFire 2.0 TCF/LEF Reporter lentivirus was purchased from System Biosciences (Palo Alto, CA). Human *RNF43* WT, 117 fs, and 659 fs cDNA subcloned into separate pMSCV expression retroviral vectors were purchased from Vectorbuilder (Chicago, IL). Human *RNF43* I48T was subcloned from human *RNF43* WT using a KOD-Plus-Mutagenesis Kit (TOYOBO, Osaka, Japan). A *CTAG1B*-subcloned pMSCV expression retroviral vector was purchased from Vectorbuilder. A lentiviral vector carrying a short hairpin RNA (shRNA) sequence to knock down murine *Rnf43* expression (sh-*Rnf43*) was purchased from Vectorbuilder. Viral vectors were transfected into packaging cells using the Lipofectamine 3000 Reagent (Thermo Fisher Scientific, Waltham, MA). After 48 h, the supernatant was concentrated and transfected into each cell line.

### Reverse transcription quantitative PCR

Total RNA was reverse transcribed into cDNA using PrimeScript RT Master Mix (TaKaRa), and reverse transcription quantitative PCR (RT-qPCR) was performed using TB Green Premix Ex Taq II (TaKaRa) according to the manufacturer’s instructions. Human *GAPDH* or murine *Gapdh* was used as an internal control. *Atf3* and *Ccl4* gene expression levels were measured after cells were treated with 10 ng/mL murine Wnt3a protein for 24 h. The experiments were performed in triplicate. The primers used are listed in Table S2.

### Luciferase assay

Each human *RNF43* mutation was introduced into HEK293T cells with the pGreenFire 2.0 TCF/LEF Reporter. After treatment with a human Wnt3a protein at 500 ng/mL for 24 h, luciferase activities were measured using the ONE-Step^TM^ Luciferase Assay System (System Biosciences). The luciferase activities of mock, human *RNF43* WT, human *RNF43* 117 fs, human *RNF43* 659 fs, and human *RNF43* I48T-overexpressing HEK293T cells were compared. All experiments were repeated independently in triplicate.

### Western blotting

After treatment with 10 ng/mL murine Wnt3a for 24 h, subconfluent cells were washed with phosphate-buffered saline buffer and harvested using 1% sodium dodecyl sulphate (SDS). Whole-cell lysates were separated using SDS-PAGE and blotted onto a polyvinylidene fluoride membrane. Post-blocking, the membrane was probed with a primary antibody. After rinsing twice with tris-buffered saline buffer, the membrane was incubated with a horseradish peroxidase-conjugated secondary antibody and washed, followed by visualisation and quantification using an ECL detection system and a ChemiDoc imaging system (Bio-Rad, Hercules, CA). Antibody specific for nonphosphorylated (active) β-catenin (D13A1) was purchased from Cell Signaling Technology. Antibody against β-actin (A5441) was purchased from Sigma–Aldrich (St. Louis, MO).

### In vivo animal models

Female Balb/c mice (6–8 weeks old) were purchased from SLC Japan (Shizuoka, Japan). C57BL/6J-Prkdc<scid>/Rbrc mice (SCID; RBRC01346) were provided by RIKEN BRC (Tsukuba, Japan) through the National BioResource Project of the MEXT/AMED, Japan. CT26 (1 × 10^6^), RENCA (2 × 10^6^), or A20 cells (4 × 10^6^) were inoculated subcutaneously, and tumour volume was monitored every 3 days. The mean of the long and short diameters was used to generate tumour growth curves. The mice were grouped when the tumour volume reached ~100 mm^3^ (Day 0), and an anti-PD-1 mAb (200 μg/mouse) or a control mAb was administered intraperitoneally every 3 days thereafter (three times total). The tumours were harvested 14 days post-tumour cell inoculation and evaluated using immunohistochemistry (IHC). All in vivo experiments were performed at least twice (*n* = 4–6 per group). All mice were maintained under specific pathogen-free conditions in the Institute of Biophysics animal facility.

### TCGA dataset analysis

WES and RNA-seq data for each cancer type were acquired from the TCGA database published in cBioPortal (https://www.cbioportal.org/) [39, 40]. The TMB was defined as the sum of the total number of nonsynonymous single-nucleotide variants (SNVs) and fs mutations. Peptide sequence information was obtained from UniProt (https://www.uniprot.org/) [41]. HLA typing data for each patient was available at TCIA (https://tcia.at/home) [42]. Cancer types with no HLA data and haematological malignancies were excluded from the analyses. The affinity of abnormal peptides for HLA class I molecules in individual tumours was predicted using NetMHCpan 4.0, and the peptides in the top two percentiles for affinity were defined as neoantigens. We defined the genes listed in the Cancer Gene Census (https://cancer.sanger.ac.uk/census) of COSMIC (https://cancer.sanger.ac.uk/cosmic) as “driver genes” and the others as “passenger genes” (Table S3) [43]. The average expression of *CD8A*, *GZMA*, and *PRF1* was computed from the RNA-seq data of the same patients to derive an “immune activity score”. The correlations between the TMB, driver/passenger mutational burden, total neoantigen load or neoantigen load derived from driver/passenger mutations, and the immune activity score were compared by computing Pearson’s correlation coefficient (R). The analysis was conducted on cases for which the TMB and predicted neoantigen loads were within the 95% confidence interval.

### Statistics

GraphPad Prism 9 (GraphPad Software, San Diego, CA) was utilised for statistical analyses. Frequencies were compared between groups using Fisher’s exact test. The relationships of continuous variables between or among groups were compared using *t*-test or one-way analysis of variance (ANOVA), respectively. The relationships among tumour volume curves were compared using two-way ANOVA. Overall survival was defined as the time from the date of surgery to that of death from any cause. The log-rank test was used to compare Kaplan–Meier curves. The correlations between two variables were evaluated by computing Pearson’s correlation coefficient (R). For multiple comparisons testing, Bonferroni corrections were employed. All tests were two-tailed, and *P* values < 0.05 were considered statistically significant.

## Results

### Neoantigenic *RNF43* mutations that are frequently observed in MSI-H colorectal cancer do not consistently induce an inflamed TME

**Fig. 1:**
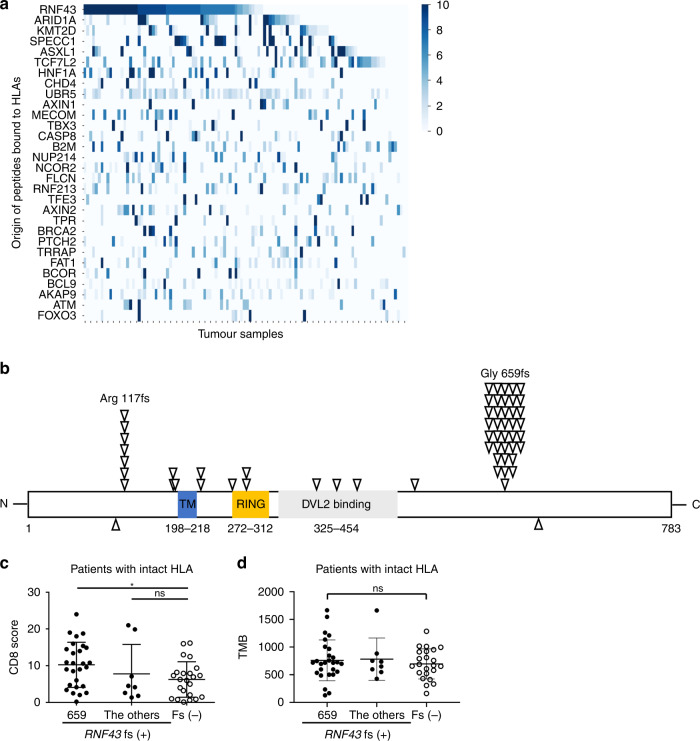
*RNF43* frameshift mutations as predicted neoantigens and immune status in the high-frequency microsatellite instability cohort. **a** Predicted neoantigens in our high-frequency microsatellite instability (MSI-H) cohort. Eighty-eight patients with MSI-H colorectal cancer who underwent surgical resection were enrolled in this study. Whole-exon sequencing (WES), RNA-sequencing, and long-read *HLA* sequencing were performed with the resected tumour samples. The NetMHCpan algorithm (version 4.0) was used to predict possible neoantigens from WES, RNA-sequencing, and long-read *HLA* sequencing data. **b** Distributions of *RNF43* frameshift (fs) mutations. All *RNF43* fs mutations derived from 88 MSI-H colorectal cancer samples are depicted with arrows. **c** Comparison of CD8^+^ T cell infiltration according to the *RNF43* status in patients with intact HLA. Summary of CD8^+^ T cell infiltration in patients with the *RNF43* 659 fs mutation, with the other fs mutations, and without any fs mutations is depicted. **d** Comparison of tumour mutational burden (TMB) according to the *RNF43* status in patients with intact HLA. The summary of TMB in patients with the *RNF43* 659 fs mutation, with the other fs mutations, and without any fs mutations is depicted. One-way ANOVA with the Bonferroni correction is used in (**c**) and (**d**) for statistical analyses. The means and SDs are depicted. *P < 0.05; ns not significant.

In this study, we evaluated 88 MSI-H colorectal cancer patients using WES, RNA-sequencing, and *HLA* long-read sequencing data (Table S1) [24]. We predicted neoantigens using NetMHCpan 4.0 and identified that peptides derived from driver gene *RNF43* fs mutations frequently became neoantigens (47 patients; Arg117fs, 7; Gly659fs, 37) (Fig. 1a, b) [37]. Although we reported that patients with HLA abnormalities had noninflamed TME and that the abnormalities were related to neoantigens, there were a considerable number of patients with intact HLA [24]. Focusing on this HLA-intact population (*n* = 58), tumours with *RNF43* fs mutations had significantly higher CD8^+^ T cell infiltration than those without it (Fig. S1A), which seems to be consistent with previous studies demonstrating that neoantigens induce an inflamed TME [9–11]. Alternatively, when *RNF43* fs mutations were divided, patients with the *RNF43* 659 fs mutation were associated with more CD8^+^ T cell infiltration than those without *RNF43* fs mutations; however, patients with the other *RNF43* fs mutations, such as the 117 fs mutation, were not (Fig. 1c). With regard to TMB, there was no significant correlation with CD8^+^ T cell infiltration or the *RNF43* fs mutations (Figs. 1d and S1B). These results suggest that not all *RNF43* fs mutations result in an inflamed TME despite being common neoantigens.

### *RNF43* fs loss-of-function mutations activate the WNT/β-catenin signalling pathway

**Fig. 2:**
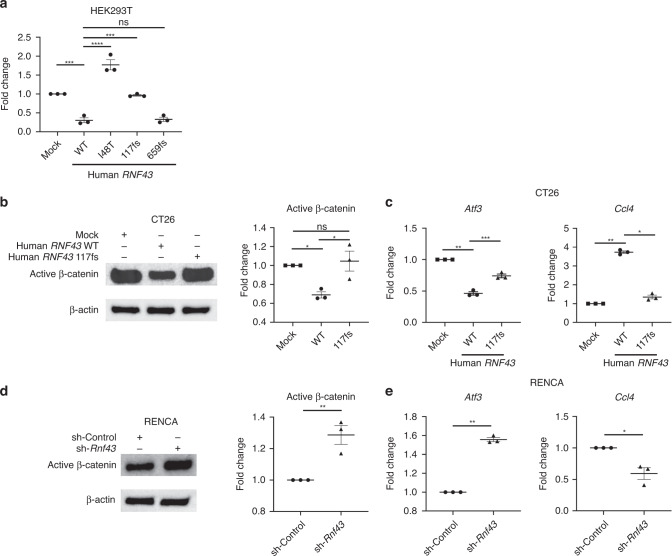
*RNF43* 117 fs loss-of-function mutation and WNT/β-catenin signalling pathway. **a** Luciferase reporter assay. The luciferase activity in each human *RNF43*-overexpressing TCF/LEF Reporter-HEK293T cell line treated with human Wnt3a was analysed. The fold changes determined by comparison to mock-overexpressing control cells are presented. **b** Western blotting. Human *RNF43*-overexpressing CT26 cells treated with murine Wnt3a were analysed using western blotting. Each band was quantified, and the fold change was computed by comparison to mock-overexpressing control cells. Representative blots (left) and the corresponding summary (right) are depicted. β-actin was used as an internal control. **c** Atf3 and Ccl4 gene expression in CT26 cells. Gene expression post-treatment with murine Wnt3a was analysed using RT-qPCR. Murine *Gapdh* was used as an internal control. The fold changes determined by comparison to mock-overexpressing control cells are presented. **d** Western blotting. *Rnf43*-knockdown (sh-*Rnf43*) RENCA cells treated with murine Wnt3a were analysed using western blotting. Each band was quantified, and the fold change was computed by comparison to sh-Control cells. Representative blots (left) and the corresponding summary (right) are depicted. β-actin was used as an internal control. **e** Atf3 and Ccl4 gene expression in RENCA cells. Gene expression post-treatment with murine Wnt3a was analysed using RT-qPCR. The fold changes determined by comparison to sh-Control cells are presented. All in vitro experiments were performed in triplicate. One-way ANOVA with the Bonferroni correction is used in (**a**–**c**), and *t*-test is used in (**d**) and (**e**) for statistical analyses. The means and SEMs are depicted. *P < 0.05; **P < 0.01; ***P < 0.001; **** < 0.0001; ns not significant.

Considering the diverse TMEs resulting from *RNF43* fs mutations in our cohort, we analysed the functions of *RNF43* fs mutations. RNF43 is a single-chain, transmembrane E3 ubiquitin ligase comprising 783 amino acids (Fig. 1b). The binding and ubiquitination of the WNT receptor, involving the RING domain, are mapped to the N-terminus, followed by the Disheveled-2 (DVL2) binding domain. No role has been assigned to the C-terminal extension. RNF43 negatively regulates the WNT/β-catenin signalling pathway and activates the signalling pathway in the presence of loss-of-function mutations [44]. We generated HEK293T cell lines with a TCF/LEF luciferase reporter (TCF/LEF Reporter-HEK293T cells) overexpressing human *RNF43* wild-type (WT); I48T, a representative WNT/β-catenin hyperactivating mutation [44]; 117 fs; or 659 fs (Fig. S2A). These cell lines were used to evaluate the activity of the WNT/β-catenin signalling pathway. We observed that *RNF43* WT and *RNF43* 659 fs suppressed the WNT/β-catenin signalling pathways, whereas *RNF43* 117 fs did not (Fig. 2a), suggesting that *RNF43* 117 fs is a loss-of-function mutation, as previously reported [34, 45, 46]. Furthermore, WNT/β-catenin signalling pathway activation was observed in patients with the *RNF43* fs mutations other than 659 fs by gene set enrichment analysis (GSEA) of the RNA-sequencing data for the HLA-intact population in our MSI-H cohort (Fig. S3A). In addition, patients with *RNF43* fs mutations, except 659 fs, had significantly shorter overall survival (OS) compared with patients with the other mutations (Fig. S3B and Table S4).

### *RNF43* fs loss-of-function mutation reduces CCL4 levels by activating the WNT/β-catenin signalling pathway

A previous report demonstrated that WNT/β-catenin signalling pathway activation induces a noninflamed TME via ATF3/CCL4 [29]. The transcriptional repressor ATF3 suppresses CCL4 and prevents effector CD8^+^ T cell infiltration by reducing dendritic cell (DC) infiltration [29]. Therefore, to evaluate the impact of *RNF43* fs mutations on TME, we examined WNT/β-catenin signalling pathway activation and *Atf3* and *Ccl4* expression using murine cell lines. We used the human *RNF43* WT- or 117fs-overexpressing CT26 (murine colorectal cancer) cell line, as this cell line had low mouse *Rnf43* expression (Fig. S2B, C). Western blotting analyses demonstrated that active β-catenin was suppressed in human *RNF43* WT-overexpressing CT26 cells, but not in human *RNF43* 117fs-overexpressing cells (Fig. 2b). Furthermore, the human *RNF43* 117 fs loss-of-function mutation led to higher *Atf3* and lower *Ccl4* expression than human *RNF43* WT (Fig. 2c). In addition, we performed mouse *Rnf43* knockdown using shRNA in RENCA and A20 cell lines because these cell lines had high *Rnf43* expression (Fig. S2B, D). *Rnf43* knockdown increased active β-catenin expression in both cell lines, resulting in higher *Atf3* and lower *Ccl4* expression (Figs. 2d, e, S2E, F). Collectively, these results indicate that *RNF43* fs loss-of-function mutations activate the WNT/β-catenin signalling pathway, leading to a reduction in the level of CCL4, a crucial chemokine for the inflamed TME.

### *RNF43* loss-of-function mutations induce a noninflamed TME and resistance to PD-1 blockade even if they produce neoantigens

**Fig. 3:**
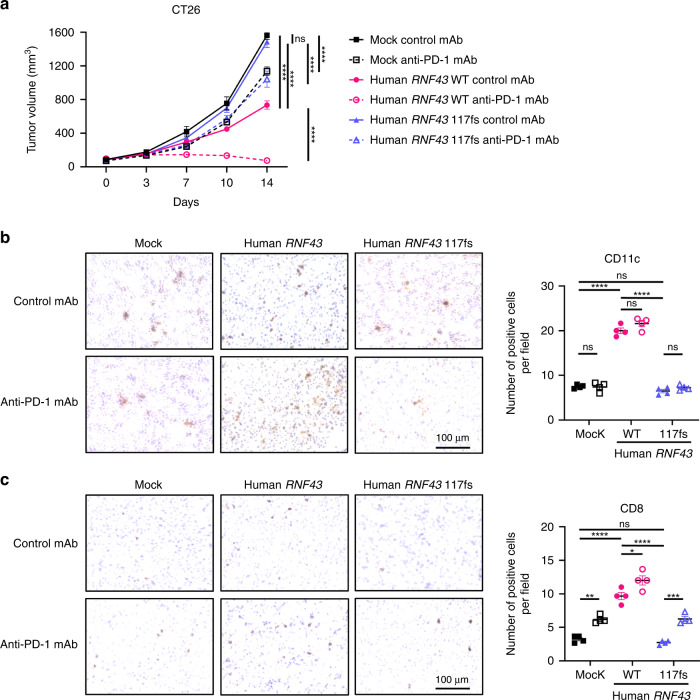
*RNF43* 117fs loss-of-function mutation and antitumor immunity in a mouse model. **a** Tumour growth of human *RNF43*-overexpressing tumours. CT26 cells (1 × 10^6^) were inoculated subcutaneously, and tumour volume was monitored every 3 days. The means of the long and short diameters were used to generate tumour growth curves. The mice were grouped when the tumour volume reached ~100 mm^3^ (Day 0), and an anti-PD-1 monoclonal antibody (mAb) or a control mAb was administered intraperitoneally every 3 days thereafter for three times total (*n* = 6 per group). **b**, **c** Immunohistochemistry (IHC) for CD11c (**b**) and CD8 (**c**). Tumours were harvested 14 days post-tumour cell inoculation for IHC. The average count of three fields (400×) for each tumour is used for statistical analysis. Representative staining figures (left) and the corresponding summaries (right) are depicted. All in vivo experiments were performed in duplicate, and similar results were obtained. Two-way ANOVA with the Bonferroni correction is used in (**a**), and one-way ANOVA with the Bonferroni correction is used in (**b**) and (**c**) for statistical analyses. The means and SEMs are depicted. *P < 0.05; **P < 0.01; ***P < 0.001; ****P < 0.0001; ns not significant.

**Fig. 4:**
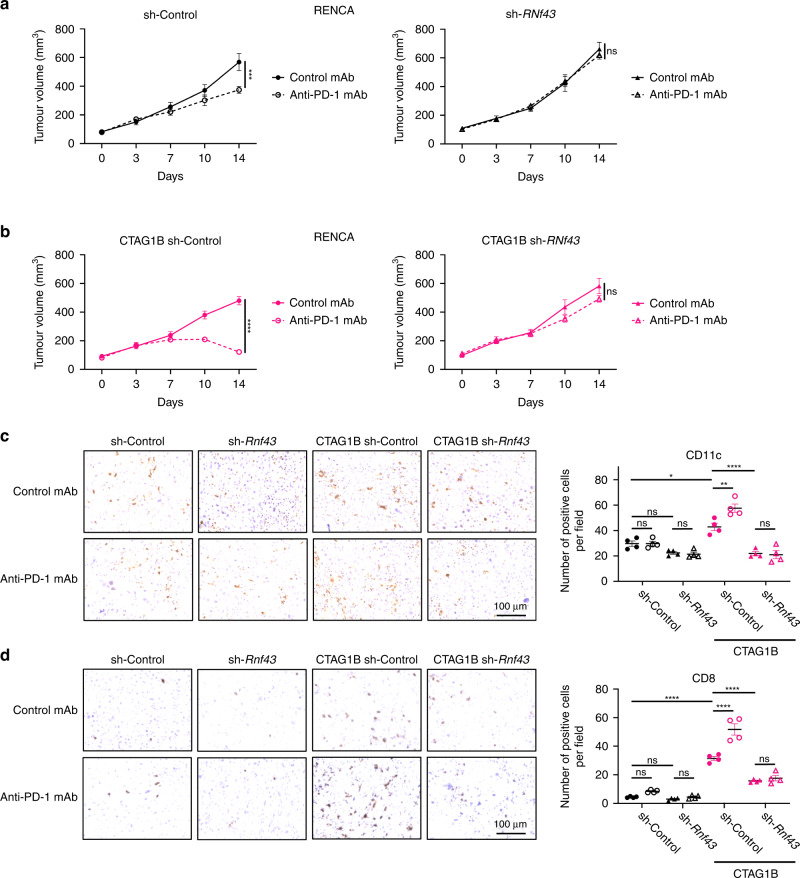
*Rnf43* knockdown and resistance to PD-1 blockade in mouse models. **a**, **b** Efficacy of PD-1 blockade against RENCA (**a**) or *CTAG1B*-overexpressing RENCA (**b**) tumours. RENCA cells (2 × 10^6^) were inoculated subcutaneously. Subsequent steps were performed as described in Fig. 3 (*n* = 5 per group). **c**, **d** IHC for CD11c (**c**) and CD8 (**d**). Tumours were harvested 14 days post-tumour cell inoculation for IHC. The average count of three fields (400×) for each tumour was used for statistical analysis. Representative staining figures (left) and the corresponding summaries (right) are depicted. All in vivo experiments were performed in duplicate, and similar results were obtained. Two-way ANOVA is used in (**a**) and (**b**), and one-way ANOVA with the Bonferroni correction is used in (**c**) and (**d**) for statistical analyses. The means and SEMs are depicted. *P < 0.05; **P < 0.01; ***P < 0.001; ****P < 0.0001; ns not significant.

We explored the impact of *RNF43* loss-of-function mutations on the therapeutic effects of ICIs and TMEs using mouse models. We used the human *RNF43* WT or 117fs-overexpressing CT26 cell lines, and compared with control tumours, human RNF43 WT-overexpressing tumours demonstrated reduced growth and improved response to an anti-PD-1 mAb (Fig. 3a). Conversely, human *RNF43* 117fs-overexpressing tumours grew similarly to control tumours, and PD-1 blockade was less effective against these tumours than against human *RNF43* WT-overexpressing tumours (Fig. 3a). These effects were not observed in immunodeficient mice (Fig. S4A). IHC revealed that human *RNF43* 117fs-overexpressing tumours had a lesser DC and CD8^+^ T cell infiltration than human *RNF43* WT-overexpressing tumours (Fig. 3b, c), which is consistent with a previous study and the results of our in vitro analysis [29]. Similarly, *Rnf43*-knockdown tumours (RENCA), generated using shRNA, were resistant to PD-1 blockade (Fig. 4a). Previous studies have demonstrated that NY-ESO-1 is a neoantigen in Balb/c mice [47, 48]; therefore, we generated *CTAG1B* (encoding NY-EOS-1)-overexpressing cell lines (RENCA and A20 cell lines) to mimic neoantigen-rich models. Accordingly, *CTAG1B*-overexpressing tumours showed improved response to PD-1 blockade compared with control tumours (Figs. 4a, b, and S4B). However, *Rnf43* knockdown abolished the efficacy against *CTAG1B*-overexpressing tumours (Figs. 4b and S4B). Efficacy against *CTAG1B*-overexpressing tumours was not observed in immunodeficient mice (Fig. S4C). IHC demonstrated that *Rnf43* knockdown impaired DC and CD8^+^ T cell infiltration even in *CTAG1B*-overexpressing tumours (Fig. 4c, d). Overall, *RNF43* loss-of-function mutations could induce a noninflamed TME and resistance to PD-1 blockade even if they produced neoantigens.

### Neoantigens derived from passenger rather than driver gene mutations are related to the inflamed TME

**Fig. 5:**
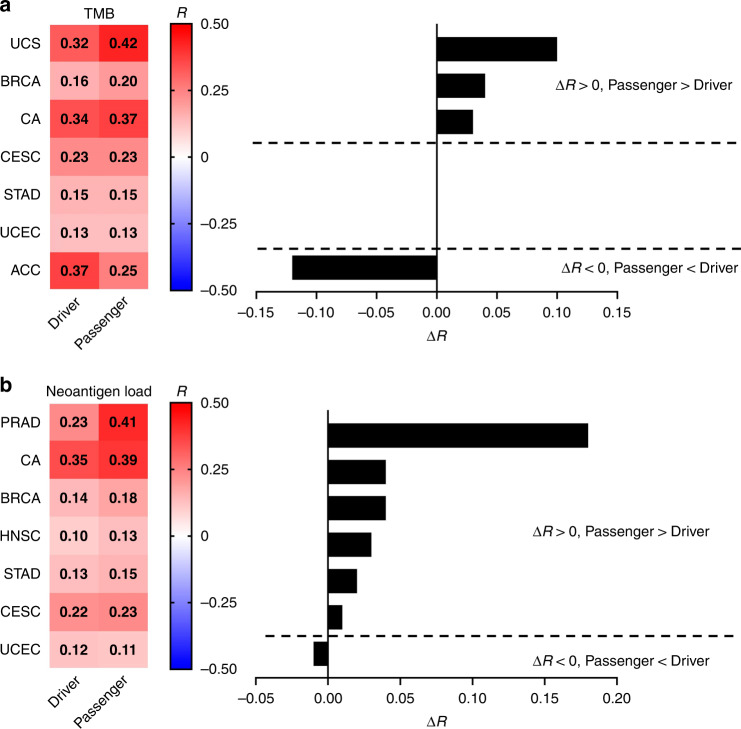
Correlations between driver or passenger gene mutations and immune responses in TCGA datasets. **a** The heatmap for Pearson’s correlation coefficients (R) between the driver/passenger mutational burden and immune activity scores (left) and the difference in R (ΔR) for each cancer type (right) are depicted. **b** The heatmaps for R between the neoantigen loads derived from driver/passenger mutations and immune activity scores (left) and ΔR for each cancer type (right) are depicted. Cancer types with *P* values < 0.05 for either driver or passenger mutations are presented. Bold R numbers indicate *P* values < 0.05. UCS uterine carcinosarcoma, BRCA breast invasive carcinoma, CA colorectal adenocarcinoma, CESC cervical squamous cell carcinoma, STAD stomach adenocarcinoma, UCEC uterine corpus endometrial carcinoma, ACC adrenocortical carcinoma, PRAD prostate adenocarcinoma, HNSC head and neck squamous cell carcinoma.

Considering that several driver gene mutations, in addition to *RNF43* mutations, have been reported to have immunosuppressive functions [25–30], we compared the effects of neoantigens derived from driver and passenger gene mutations on immune responses. We defined “driver genes” as genes listed in the Cancer Gene Census of COSMIC and the other genes as “passenger genes” (Table S3). We computed the average expression of *CD8A* and cytotoxicity-related genes (*GZMA* and *PRF1*) to produce an “immune activity score” based on a previous report [11] and evaluated the correlations between the TMB or predicted neoantigen loads and this score using TCGA datasets. As previously reported, there were certain positive correlations between the TMB or predicted neoantigen loads and immune activity scores in several cancer types (Table S5) [11]. When the driver and passenger mutations were evaluated separately, the latter had more positive and significant correlations with immune activity scores (Fig. 5, Tables S6 and 7).

## Discussion

The need to identify biomarkers to predict the response to ICIs is imperative in clinical settings. Even though several basic and clinical studies on biomarkers have been conducted, we still cannot predict the response accurately. The TMB can be a predictive biomarker as it reflects the number of neoantigens [10, 12, 13]. However, the neoantigen theory, which states that neoantigens induce an inflamed TME, is not always accurate as the correlation between neoantigens and the TME immune status is not always substantial, and the effect of ICI therapy is not well predicted [17, 18]. In this study, we demonstrated that the functions of genetic variants could paradoxically induce a noninflamed TME even if the variants served as neoantigens (“paradoxical neoantigenic mutations”). Considering that several driver gene mutations reportedly play substantial roles in antitumor immunity evasion as well as in cell proliferation and cell death [25–30], we should evaluate the qualities in addition to quantities of neoantigenic mutations.

We evaluated 88 MSI-H colorectal cancer patients and identified that fs mutations in the driver gene *RNF43* were shared neoantigens among patients, as previously reported [32]. Tumours with neoantigens derived from these *RNF43* fs mutations tended to have an inflamed TME, which is consistent with the neoantigen theory. However, the TME demonstrated differences among the fs mutations in our study. *RNF43* is a tumour suppressor gene that suppresses the WNT/β-catenin signalling pathway, and its RING domain plays a substantial role in its function [49]. Although fs mutations generally result in a loss of function, some previous studies have demonstrated that functions differ among *RNF43* fs mutations [34, 35, 45, 46]. We demonstrated that *RNF43* 117fs, a common fs mutation in the N-terminal side of the RING domain, is a loss-of-function mutation that activates the WNT/β-catenin signalling pathway [33–35, 45]. Conversely, *RNF43* 659fs, which is in the C-terminal side of the RING domain, was comparable to *RNF43* WT in suppressing the WNT/β-catenin signalling pathway [34, 35, 45, 46]. Previous reports have demonstrated that WNT/β-catenin signalling pathway suppresses immunity [26, 29]. Accordingly, the WNT/β-catenin signalling pathway was not activated via the 659fs mutation, resulting in an inflamed TME owing to neoantigens. Alternatively, the WNT/β-catenin signalling pathway activation resulting from loss-of-function fs mutations led to a noninflamed TME even in the presence of neoantigens and resistance to PD-1 blockade. To our knowledge, this is the first study demonstrating that neoantigenic *RNF43* mutations (i.e. 117 fs) can induce a noninflamed TME by WNT/β-catenin signalling pathway activation because of the loss of function.

We previously reported that even when somatic mutations increased, tumour cells could escape antitumor immunity by increasing the activity of immunosuppressive signalling pathways such as the WNT/β-catenin pathway [28, 30]. We also reported that tumour cells evade antitumor immunity by mutating HLA genes and that driver gene mutations play significant roles in not only proliferation and survival but also antitumor immunity evasion [24, 27, 28]. Moreover, the present study demonstrates that some of the neoantigenic mutations, particularly driver mutations, can paradoxically act as antitumor immunity suppressors through the original gene functions. These observations could explain the weak correlation with the immune status of the TME and the inadequacy in predicting the effects of ICIs [24, 27, 28, 30].

Several reports have demonstrated the presence of *RNF43* fs mutations in MSI-H colorectal cancer [32–35]. In addition, the relationship between *RNF43* mutations and the WNT/β-catenin signalling pathway has been reported [34, 35, 45, 46]. However, these studies did not focus on antitumor immunity and PD-1 blockade-mediated efficacy based on the neoantigenic *RNF43* mutations. Previous studies have reported that WNT/β-catenin signalling pathway activation induces a noninflamed TME [29, 30]; however, this study, to our knowledge, has for the first time demonstrated that neoantigenic *RNF43* mutations (i.e. 117 fs) can induce the noninflamed TME by WNT/β-catenin signalling pathway activation because of the loss of function. These detailed analyses are limited to MSI-H cancer as there are few shared driver genes and more individualised mutations reported as candidate genes for neoantigens [8, 50]. In addition, there are few reports of common neoantigens in other cancer types [32], making such analyses difficult. Conversely, using TCGA dataset analyses, we demonstrated that passenger rather than driver gene mutations were related to the inflamed TME. Thus, even if such functional driver gene mutations become neoantigens, patients with these neoantigens could have a noninflamed TME because of gene functions. These findings suggest the need to evaluate the qualities as well as quantities of neoantigenic mutations. To validate these results, further research is warranted.

In summary, we identified “paradoxical neoantigenic mutations” that could induce a noninflamed TME owing to their original gene function, despite deriving neoantigens. We propose the need to assess the qualities as well as the quantities of neoantigenic gene mutations, particularly driver gene mutations, as predictive biomarkers for ICI response. Further studies using large cohorts are warranted to determine the clinical applicability of our results.

## Supplementary information

Figure S1

Figure S2

Figure S3

Figure S4

Table S1

Table S2

Table S3

Table S4

Table S5

Table S6

Table S7

## Data Availability

The data that support the results of this study are available from the corresponding author, YT, upon reasonable request. Raw sequencing data were deposited in the Japanese Genotype-Phenotype Archive (http://trace.ddbj.nig.ac.jp/jga), which is hosted by the DNA Data Bank (10.1093/nar/gku1120) of Japan, under accession number JGAS00000000113 (NBDC number: hum0094).
