# Supplementary material for: Somatic mutations can induce a noninflamed tumour microenvironment via their original gene functions, despite deriving neoantigens
Source: Br J Cancer. 2023 Feb 2;128(6):1166–75. doi: 10.1038/s41416-023-02165-6 (PMC10006227; doi:10.1038/s41416-023-02165-6)
Supplement: Supplementary file 1 — Figure S1 [file 41416_2023_2165_MOESM1_ESM.pdf]

Figure S1. Corelation among CD8+ T cell infiltration, RNF43 gene status, and TMB.

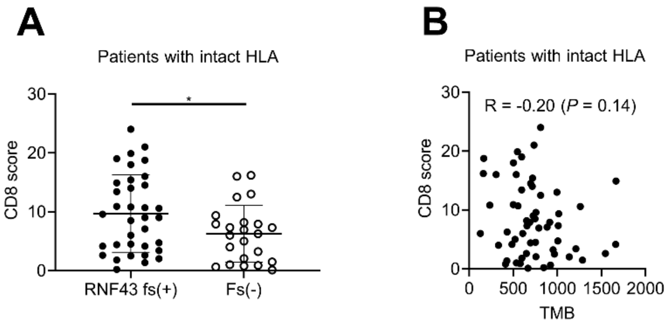

A. Comparison of CD8$^+$ T cell infiltration according to the *RNF43* status in patients with intact HLA. The summary of CD8$^+$ T cell infiltration in patients with and without *RNF43* fs mutations is depicted.

B. Correlation between TMB and CD8$^+$ T cell infiltration in patients with intact HLA. Pearson's correlation coefficient (R) is depicted.

T test was used in (A), and one-way ANOVA with the Bonferroni correction was used in (C) for statistical analyses. The means and SDs are depicted. *$P < 0.05$; ns, not significant.
