# Supplementary material for: Somatic mutations can induce a noninflamed tumour microenvironment via their original gene functions, despite deriving neoantigens
Source: Br J Cancer. 2023 Feb 2;128(6):1166–75. doi: 10.1038/s41416-023-02165-6 (PMC10006227; doi:10.1038/s41416-023-02165-6)
Supplement: Supplementary file 2 — Figure S2 [file 41416_2023_2165_MOESM2_ESM.pdf]

Figure S2. Additional *in vitro* experiments.

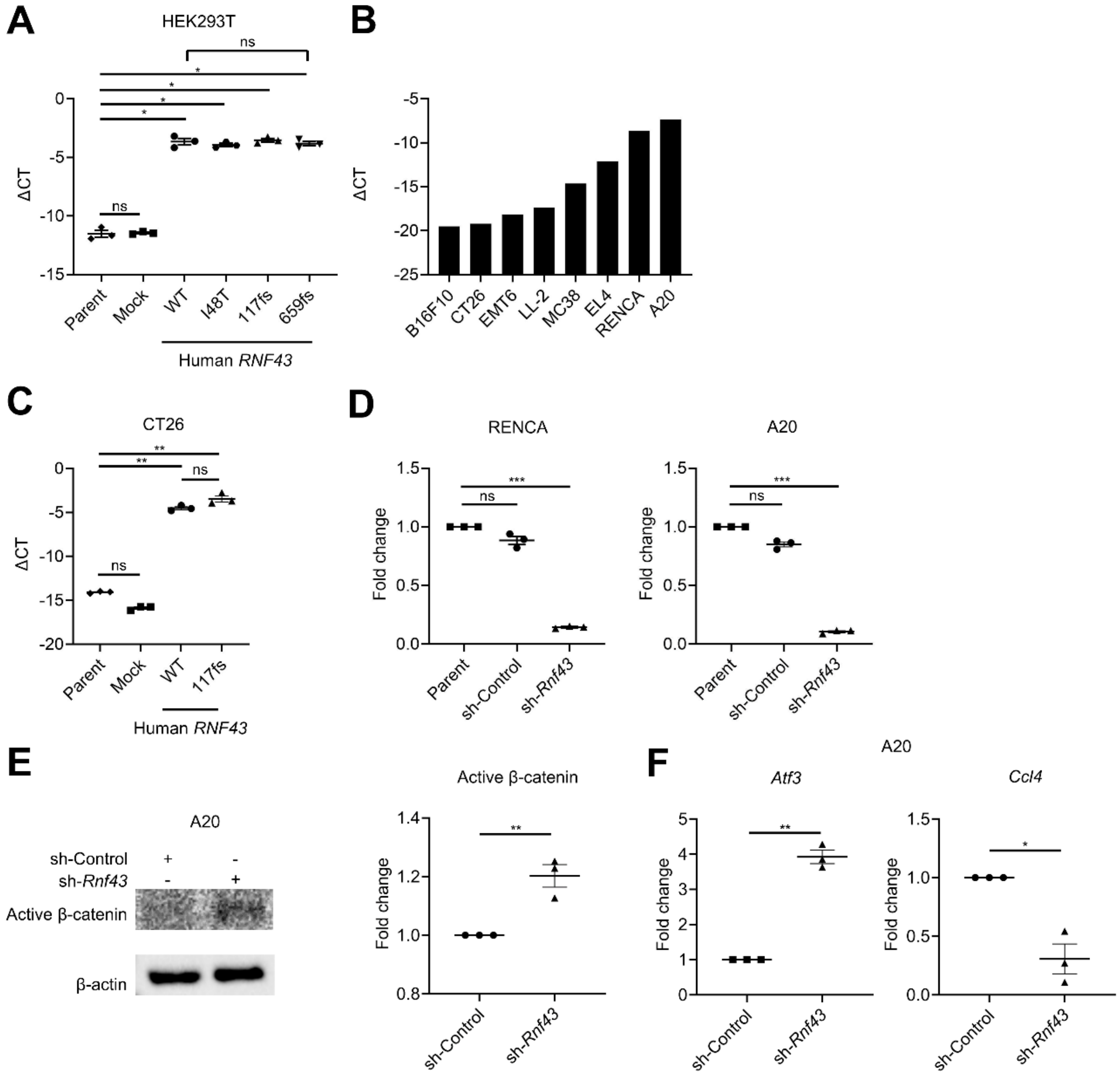

Gene expression and protein expression were evaluated with RT–qPCR and western blotting, respectively.

A. Human *RNF43* gene expression in each TCF/LEF Reporter-HEK293T cell line. Mock and each human *RNF43* was transduced, and gene expression was evaluated with RT–qPCR. Human *GAPDH* was used as an internal control.

B. Rnf43 gene expression in murine cell lines. Gene expression was evaluated with RT–qPCR. Murine *Gapdh* was used as an internal control.

C. Human RNF43 gene expression in each *RNF43*-overexpressed CT26 cell line. Mock and each human *RNF43* was transduced, and gene expression was evaluated with RT–qPCR. Murine *Gapdh* was used as an internal control.

D. *Rnf43* knockdown using shRNA in RENCA and A20 cell lines. Murine *Gapdh* was used as an internal control. The fold changes determined by comparison to parental cells are depicted.

E. Western blotting. *Rnf43*-knockdown A20 cells treated with murine Wnt3a were evaluated by western blotting. Each band was quantified, and the fold change was calculated by comparison to sh-Control cells. Representative blots (left) and the corresponding summary (right) are depicted. β-actin was used as an internal control.

F. Atf3 and Ccl4 gene expression in A20 cells. Gene expression after treatment with murine Wnt3a was evaluated with RT–qPCR. Murine *Gapdh* was used as an internal control. The fold changes determined by comparison to sh-Control cells are depicted.

The *in vitro* experiments in (A), (C)-(F) were performed in triplicate. One-way ANOVA with the Bonferroni correction was used in (A), (C) and (D), and t tests were used in (E) and (F) for statistical analyses. The means and SEMs are depicted. *$P < 0.05$; **$P < 0.01$; ***$P < 0.001$; ns, not significant.
