# Supplementary material for: Somatic mutations can induce a noninflamed tumour microenvironment via their original gene functions, despite deriving neoantigens
Source: Br J Cancer. 2023 Feb 2;128(6):1166–75. doi: 10.1038/s41416-023-02165-6 (PMC10006227; doi:10.1038/s41416-023-02165-6)
Supplement: Supplementary file 3 — Figure S3 [file 41416_2023_2165_MOESM3_ESM.pdf]

Figure S3. GSEA and overall survival.

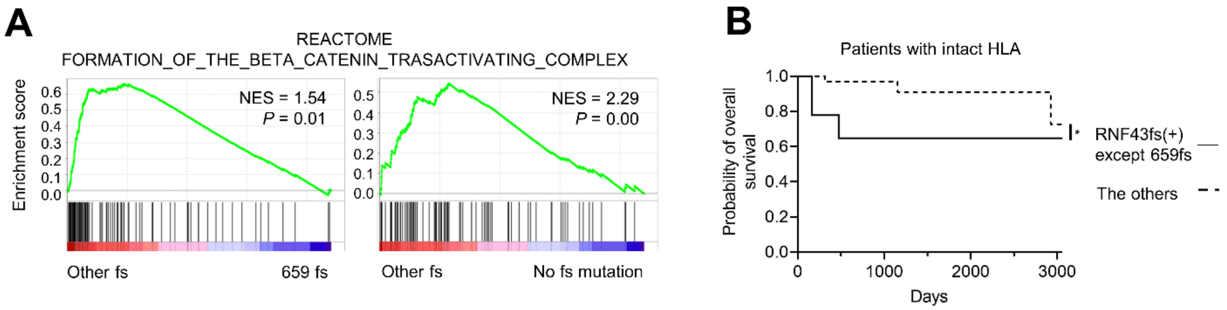

A. GSEA. We evaluated RNA-sequencing data from 55 MSI-H colorectal cancers with intact HLA using GSEA according to the *RNF43* status.

B. Kaplan–Meier curves for overall survival in the HLA-intact population. Overall survival between the patients with and without *RNF43* fs mutations, except the 659fs mutation was compared.

The log-rank test was used to compare Kaplan–Meier curves. *$P < 0.05$.
