# Supplementary material for: Somatic mutations can induce a noninflamed tumour microenvironment via their original gene functions, despite deriving neoantigens
Source: Br J Cancer. 2023 Feb 2;128(6):1166–75. doi: 10.1038/s41416-023-02165-6 (PMC10006227; doi:10.1038/s41416-023-02165-6)
Supplement: Supplementary file 4 — Figure S4 [file 41416_2023_2165_MOESM4_ESM.pdf]

Figure S4. Additional *in vivo* experiments.

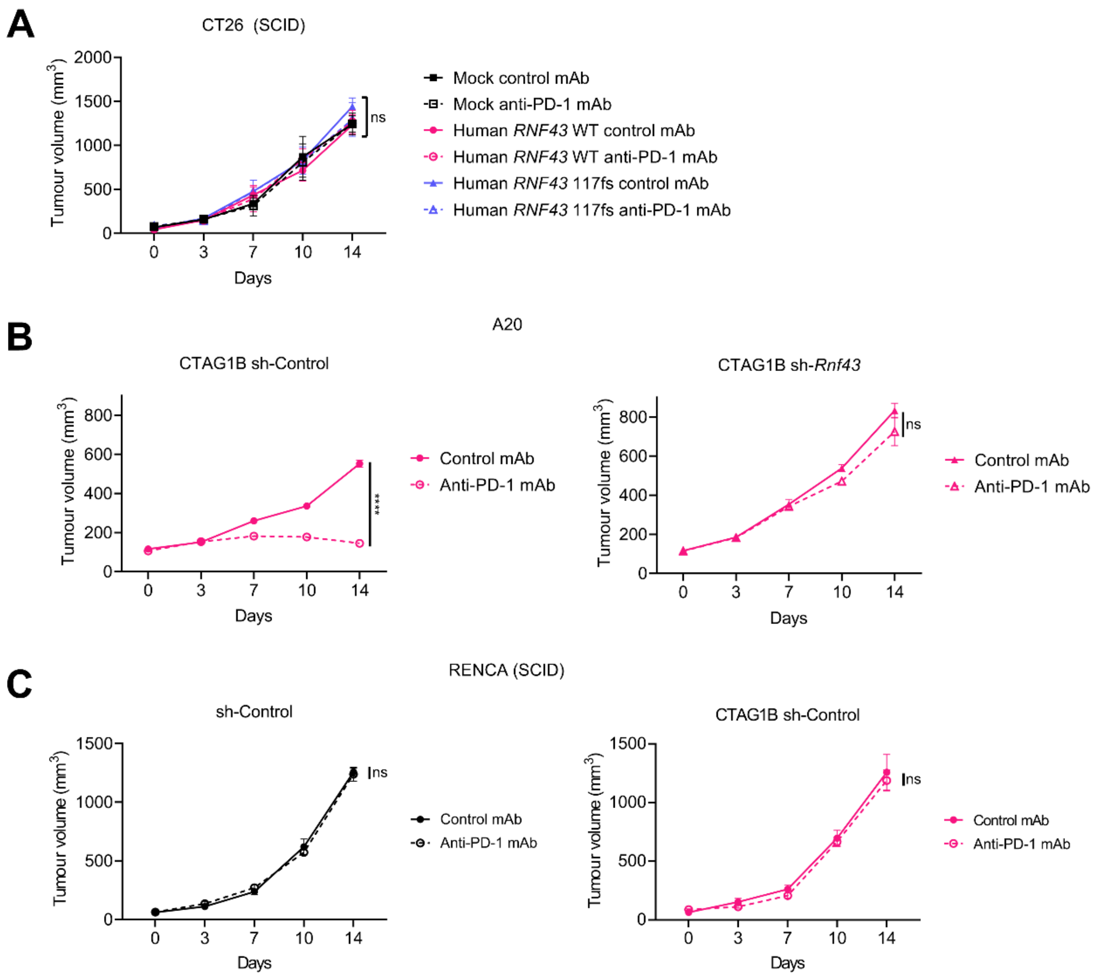

A. Efficiency of PD-1 blockade against CT26 tumours in immunodeficient SCID mice. CT26 cells (1×10^6) were inoculated subcutaneously into immunodeficient SCID mice, and subsequent steps were performed as described in Figure 3 (n = 4 per group).

B. Efficacy of PD-1 blockade against *Rnf43*-knockdown A20 tumours expressing a neoantigen. A20 cells (4×10^6) were inoculated subcutaneously. Subsequent steps were performed as described in Figure 3 (n = 4 per group).

C. Efficacy of PD-1 blockade against RENCA tumours in immunodeficient SCID mice. RENCA cells (2×10^6) were inoculated subcutaneously into immunodeficient SCID mice, and subsequent steps were performed as described in Figure 3 (n = 4 per group).

All *in vivo* experiments were performed in duplicate and produced similar results. One-way ANOVA was used for statistical analyses. The means and SEMs are depicted. ****P < 0.0001; ns, not significant.
