# Supplementary material for: Somatic mutations can induce a noninflamed tumour microenvironment via their original gene functions, despite deriving neoantigens
Source: Br J Cancer. 2023 Feb 2;128(6):1166–75. doi: 10.1038/s41416-023-02165-6 (PMC10006227; doi:10.1038/s41416-023-02165-6)
Supplement: Supplementary file 5 — Table S1 [file 41416_2023_2165_MOESM5_ESM.pdf]

Table S1. Patient characteristics.

Characteristic	Value
Age, mean ± standard deviation	71 ± 14
Male, no. (%)	36 (42%)
Site of tumour, no. (%)†	
Cecum	18 (20%)
Ascending colon	27 (31%)
Transverse colon	22 (25%)
Descending colon	4 (4.5%)
Sigmoid colon	11 (13%)
Rectum	6 (6.8%)
Invasion depth, no. (%)†§	
T0	0 (0.0%)
Tumour *in situ*	2 (2.3%)
T1	10 (11%)
T2	14 (16%)
T3	45 (51%)
T4	17 (19%)
Regional lymph nodes metastasis, no. (%)†	
NX	9 (10%)
N0	49 (57%)
N1	21 (24%)
N2	7 (8.1%)
Distant metastasis, no. (%)†§	
M0	85 (99%)
M1	1 (1.2%)
Stage, no. (%)†	
0	2 (2.3%)
I	14 (16%)
II	33 (38%)
III	28 (33%)
IV	1 (1.1%)
Not available	8 (9.1%)

†88 tumours from 86 patients with two synchronous cases; §TNM Classification of Malignant Tumours (7th edition), Union for International Cancer Control.
