# Supplementary material for: Somatic mutations can induce a noninflamed tumour microenvironment via their original gene functions, despite deriving neoantigens
Source: Br J Cancer. 2023 Feb 2;128(6):1166–75. doi: 10.1038/s41416-023-02165-6 (PMC10006227; doi:10.1038/s41416-023-02165-6)
Supplement: Supplementary file 6 — Table S2 [file 41416_2023_2165_MOESM6_ESM.pdf]

Table S2. Primers for real time RT-qPCR.

Gene name		Sequence
Human *RNF43*	Forward	TGGAAGGTGTGTTTGCTGGT
	Reverse	CGTCATCACTGGCATTGCAC
Human *GAPDH*	Forward	ACCACAGTCCATGCCATCAC
	Reverse	TACAGCAACAGGGTGGTGGA
Murine *Rnf43*	Forward	GGAGATGTCCTTACTCACATTGC
	Reverse	CTGACCAGCTCACGTCACAC
Murine *Atf3*	Forward	GCTGGAGTCAGTTACCGTCAA
	Reverse	CGCCTCCTTTTCCTCTCAT
Murine *Ccl4*	Forward	GCCCTCTCTCTCCTCTTGCT
	Reverse	GGAGGGTCAGAGCCCATT
Murine *Gapdh*	Forward	CATCACTGCCACCCAGAAGACTG
	Reverse	ATGCCAGTGAGCTTCCCGTTCAG
