# Supplementary material for: Somatic mutations can induce a noninflamed tumour microenvironment via their original gene functions, despite deriving neoantigens
Source: Br J Cancer. 2023 Feb 2;128(6):1166–75. doi: 10.1038/s41416-023-02165-6 (PMC10006227; doi:10.1038/s41416-023-02165-6)
Supplement: Supplementary file 7 — Table S3 [file 41416_2023_2165_MOESM7_ESM.pdf]

Table S3. Driver genes listed in the Cancer Gene Census (https://cancer.sanger.ac.uk/census) of COSMIC (https://cancer.sanger.ac.uk/cosmic).

Gene Symbol
A1CF
ABI1
ABL1
ABL2
ACKR3
ACSL3
ACSL6
ACVR1
ACVR2A
AFDN
AFF1
AFF3
AFF4
AKAP9
AKT1
AKT2
AKT3
ALDH2
ALK
AMER1
ANK1
APC
APOBEC3B
AR
ARAF
ARHGAP26
ARHGAP35
ARHGAP5
ARHGEF10
ARHGEF10L
ARHGEF12
ARID1A
ARID1B
ARID2
ARNT
ASPSCR1
ASXL1

ASXL2
ATF1
ATIC
ATM
ATP1A1
ATP2B3
ATR
ATRX
AXIN1
AXIN2
B2M
BAP1
BARD1
BAX
BAZ1A
BCL10
BCL11A
BCL11B
BCL2
BCL2L12
BCL3
BCL6
BCL7A
BCL9
BCL9L
BCLAF1
BCOR
BCORL1
BCR
BIRC3
BIRC6
BLM
BMP5
BMPR1A
BRAF
BRCA1
BRCA2
BRD3
BRD4
BRIP1

BTG1
BTG2
BTK
BUB1B
C15orf65
CACNA1D
CALR
CAMTA1
CANT1
CARD11
CARS
CASP3
CASP8
CASP9
CBFA2T3
CBFB
CBL
CBLB
CBLC
CCDC6
CCNB1IP1
CCNC
CCND1
CCND2
CCND3
CCNE1
CCR4
CCR7
CD209
CD274
CD28
CD74
CD79A
CD79B
CDC73
CDH1
CDH10
CDH11
CDH17
CDK12

CDK4
CDK6
CDKN1A
CDKN1B
CDKN2A
CDKN2C
CDX2
CEBPA
CEP89
CHCHD7
CHD2
CHD4
CHEK2
CHIC2
CHST11
CIC
CIITA
CLIP1
CLP1
CLTC
CLTCL1
CNBD1
CNBP
CNOT3
CNTNAP2
CNTRL
COL1A1
COL2A1
COL3A1
COX6C
CPEB3
CREB1
CREB3L1
CREB3L2
CREBBP
CRLF2
CRNKL1
CRTC1
CRTC3
CSF1R

CSF3R
CSMD3
CTCF
CTNNA2
CTNNB1
CTNND1
CTNND2
CUL3
CUX1
CXCR4
CYLD
CYP2C8
CYSLTR2
DAXX
DCAF12L2
DCC
DCTN1
DDB2
DDIT3
DDR2
DDX10
DDX3X
DDX5
DDX6
DEK
DGCR8
DICER1
DNAJB1
DNM2
DNMT3A
DROSHA
DUX4L1
EBF1
ECT2L
EED
EGFR
EIF1AX
EIF3E
EIF4A2
ELF3

ELF4
ELK4
ELL
ELN
EML4
EP300
EPAS1
EPHA3
EPHA7
EPS15
ERBB2
ERBB3
ERBB4
ERC1
ERCC2
ERCC3
ERCC4
ERCC5
ERG
ESR1
ETNK1
ETV1
ETV4
ETV5
ETV6
EWSR1
EXT1
EXT2
EZH2
EZR
FAM131B
FAM135B
FAM47C
FANCA
FANCC
FANCD2
FANCE
FANCF
FANCG
FAS

FAT1
FAT3
FAT4
FBLN2
FBXO11
FBXW7
FCGR2B
FCRL4
FEN1
FES
FEV
FGFR1
FGFR1OP
FGFR2
FGFR3
FGFR4
FH
FHIT
FIP1L1
FKBP9
FLCN
FLI1
FLNA
FLT3
FLT4
FNBP1
FOXA1
FOXL2
FOXO1
FOXO3
FOXO4
FOXP1
FOXR1
FSTL3
FUBP1
FUS
GAS7
GATA1
GATA2
GATA3

GLI1
GMPS
GNA11
GNAQ
GNAS
GOLGA5
GOPC
GPC3
GPC5
GPHN
GRIN2A
GRM3
H3F3A
H3F3B
HERPUD1
HEY1
HIF1A
HIP1
HIST1H3B
HIST1H4I
HLA-A
HLF
HMGA1
HMGA2
HMGN2P46
HNF1A
HNRNPA2B1
HOOK3
HOXA11
HOXA13
HOXA9
HOXC11
HOXC13
HOXD11
HOXD13
HRAS
HSP90AA1
HSP90AB1
ID3
IDH1

IDH2
IGF2BP2
IGH
IGK
IGL
IKBKB
IKZF1
IKZF3
IL2
IL21R
IL6ST
IL7R
IRF4
IRS4
ISX
ITGAV
ITK
JAK1
JAK2
JAK3
JAZF1
JUN
KAT6A
KAT6B
KAT7
KCNJ5
KDM5A
KDM5C
KDM6A
KDR
KDSR
KEAP1
KIAA1549
KIF5B
KIT
KLF4
KLF6
KLK2
KMT2A
KMT2C

KMT2D
KNL1
KNSTRN
KRAS
KTN1
LARP4B
LASP1
LATS1
LATS2
LCK
LCP1
LEF1
LEPROTL1
LHFPL6
LIFR
LMNA
LMO1
LMO2
LPP
LRIG3
LRP1B
LSM14A
LYL1
LYN
LZTR1
MACC1
MAF
MAFB
MALAT1
MALT1
MAML2
MAP2K1
MAP2K2
MAP2K4
MAP3K1
MAP3K13
MAPK1
MAX
MB21D2
MDM2

MDM4
MDS2
MECOM
MED12
MEN1
MET
MGMT
MITF
MLF1
MLH1
MLLT1
MLLT10
MLLT11
MLLT3
MLLT6
MN1
MNX1
MPL
MRTFA
MSH2
MSH6
MSI2
MSN
MTCP1
MTOR
MUC1
MUC16
MUC4
MUTYH
MYB
MYC
MYCL
MYCN
MYD88
MYH11
MYH9
MYO5A
MYOD1
N4BP2
NAB2

NACA
NBEA
NBN
NCKIPSD
NCOA1
NCOA2
NCOA4
NCOR1
NCOR2
NDRG1
NF1
NF2
NFATC2
NFE2L2
NFIB
NFKB2
NFKBIE
NIN
NKX2-1
NONO
NOTCH1
NOTCH2
NPM1
NR4A3
NRAS
NRG1
NSD1
NSD2
NSD3
NT5C2
NTHL1
NTRK1
NTRK3
NUMA1
NUP214
NUP98
NUTM1
NUTM2B
NUTM2D
OLIG2

OMD
P2RY8
PABPC1
PAFAH1B2
PALB2
PATZ1
PAX3
PAX5
PAX7
PAX8
PBRM1
PBX1
PCBP1
PCM1
PDCD1LG2
PDE4DIP
PDGFB
PDGFRA
PDGFRB
PER1
PHF6
PHOX2B
PICALM
PIK3CA
PIK3CB
PIK3R1
PIM1
PLAG1
PLCG1
PML
PMS1
PMS2
POLD1
POLE
POLG
POLQ
POT1
POU2AF1
POU5F1
PPARG

PPFIBP1
PPM1D
PPP2R1A
PPP6C
PRCC
PRDM1
PRDM16
PRDM2
PREX2
PRF1
PRKACA
PRKAR1A
PRKCB
PRPF40B
PRRX1
PSIP1
PTCH1
PTEN
PTK6
PTPN11
PTPN13
PTPN6
PTPRB
PTPRC
PTPRD
PTPRK
PTPRT
PWWP2A
QKI
RABEP1
RAC1
RAD17
RAD21
RAD51B
RAF1
RALGDS
RANBP2
RAP1GDS1
RARA
RB1

RBM10
RBM15
RECQL4
REL
RET
RFWD3
RGPD3
RGS7
RHOA
RHOH
RMI2
RNF213
RNF43
ROBO2
ROS1
RPL10
RPL22
RPL5
RPN1
RSPO2
RSPO3
RUNX1
RUNX1T1
S100A7
SALL4
SBDS
SDC4
SDHA
SDHAF2
SDHB
SDHC
SDHD
5-Sep
6-Sep
9-Sep
SET
SETBP1
SETD1B
SETD2
SETDB1

SF3B1
SFPQ
SFRP4
SGK1
SH2B3
SH3GL1
SHTN1
SIRPA
SIX1
SIX2
SKI
SLC34A2
SLC45A3
SMAD2
SMAD3
SMAD4
SMARCA4
SMARCB1
SMARCD1
SMARCE1
SMC1A
SMO
SND1
SNX29
SOCS1
SOX2
SOX21
SPECC1
SPEN
SPOP
SRC
SRGAP3
SRSF2
SRSF3
SS18
SS18L1
SSX1
SSX2
SSX4
STAG1

STAG2
STAT3
STAT5B
STAT6
STIL
STK11
STRN
SUFU
SUZ12
SYK
TAF15
TAL1
TAL2
TBL1XR1
TBX3
TCEA1
TCF12
TCF3
TCF7L2
TCL1A
TEC
TENT5C
TERT
TET1
TET2
TFE3
TFEB
TFG
TFPT
TFRC
TGFBR2
THRAP3
TLX1
TLX3
TMEM127
TMPRSS2
TMSB4X
TNC
TNFAIP3
TNFRSF14

TNFRSF17
TOP1
TP53
TP63
TPM3
TPM4
TPR
TRA
TRAF7
TRB
TRD
TRIM24
TRIM27
TRIM33
TRIP11
TRRAP
TSC1
TSC2
TSHR
U2AF1
UBR5
USP44
USP6
USP8
USP9X
VAV1
VHL
VTI1A
WAS
WDCP
WIF1
WNK2
WRN
WT1
WWTR1
XPA
XPC
XPO1
YWHAE
ZBTB16

ZCCHC8
ZEB1
ZFHX3
ZMYM2
ZMYM3
ZNF331
ZNF384
ZNF429
ZNF479
ZNF521
ZNRF3
ZRSR2
