# Supplementary material for: Somatic mutations can induce a noninflamed tumour microenvironment via their original gene functions, despite deriving neoantigens
Source: Br J Cancer. 2023 Feb 2;128(6):1166–75. doi: 10.1038/s41416-023-02165-6 (PMC10006227; doi:10.1038/s41416-023-02165-6)
Supplement: Supplementary file 8 — Table S4 [file 41416_2023_2165_MOESM8_ESM.pdf]

Table S4. Comparison of patient characteristics (patients with intact HLA).

**Characteristic**	**RNF43 fs mutation (+) except 659fs (N = 14)**	**Others† (N = 44)**	***P* value**
Median age (range) - year	75 ± 12	72 ± 12	0.90
Sex, no. (%)			0.37
Female	10 (71%)	25 (57%)	
Male	4 (29%)	19 (43%)	
Site of tumour§, no. (%)			> 0.99
Right	12 (86%)	35 (80%)	
Left	2 (14%)	9 (20%)	
Stage, no. (%)			0.20
0 - II	7 (59%)	27 (71%)	
III - IV	7 (50%)	11 (29%)	

†6 Patient staging data were not available. §"Right" was defined as the cecum, ascending colon and transverse colon, and "Left" was defined as the descending colon, sigmoid colon and rectum.
