# Supplementary material for: Somatic mutations can induce a noninflamed tumour microenvironment via their original gene functions, despite deriving neoantigens
Source: Br J Cancer. 2023 Feb 2;128(6):1166–75. doi: 10.1038/s41416-023-02165-6 (PMC10006227; doi:10.1038/s41416-023-02165-6)
Supplement: Supplementary file 9 — Table S5 [file 41416_2023_2165_MOESM9_ESM.pdf]

Table S5. Correlation between TMB or predicted neoantigen loads and immune activity scores.

Cancer type	TMB	*P* value	Neoantigens	*P* value
ACC	0.26	0.026	N/A	N/A
BLCA	0.080	0.12	0.18	<0.0001
BRCA	0.20	<0.0001	0.35	<0.0001
CA	0.37	<0.0001	0.39	<0.0001
CESC	0.23	0.0002	0.23	0.0001
CHOL	-0.11	0.55	N/A	N/A
ESCA	-0.12	0.12	N/A	N/A
GBM	-0.041	0.61	-0.047	0.57
HNSC	0.029	0.53	0.13	0.0060
KICH	-0.025	0.84	0.078	0.54
KIRC	0.034	0.53	0.037	0.49
KIRP	-0.061	0.32	-0.030	0.62
LGG	0.062	0.16	N/A	N/A
LIHC	0.035	0.52	-0.058	0.28
LS	0.043	0.37	0.071	0.13
LUAD	0.066	0.15	0.052	0.26
MESO	-0.29	0.0098	N/A	N/A
OV	0.041	0.56	0.034	0.63
PAAD	-0.26	0.0006	-0.21	0.0052
PCPG	-0.046	0.55	N/A	N/A
PRAD	-0.054	0.24	0.40	<0.0001
SARC	0.050	0.46	N/A	N/A
SKCM	-0.20	0.67	-0.0098	0.93
STAD	0.15	0.0026	0.15	0.0029
TGCT	-0.11	0.22	N/A	N/A
THCA	0.031	0.50	-0.062	0.18
THYM	-0.57	<0.0001	N/A	N/A
UCEC	0.13	0.0048	0.11	0.012
UCS	0.42	0.014	N/A	N/A
UVM	-0.20	0.072	N/A	N/A

ACC, adrenocortical carcinoma; BLCA, bladder urothelial carcinoma; BRCA, breast invasive carcinoma; CA, colorectal adenocarcinoma; CESC, cervical squamous cell carcinoma; CHOL, cholangiocarcinoma; ESCA, esophageal adenocarcinoma; GBM, glioblastoma multiforme; HNSC, head and neck squamous cell carcinoma; KICH, kidney chromophobe; KIRC, kidney renal clear cell carcinoma; KIRP, kidney renal papillary cell carcinoma; LGG, brain low grade glioma; LIHC, liver hepatocellular carcinoma; LS, lung squamous cell carcinoma; LUAD, lung adenocarcinoma; MESO, mesothelioma; OV, ovarian serous cystadenocarcinoma; PAAD, pancreatic adenocarcinoma; PCPG, pheochromocytoma and paraganglioma; PRAD, prostate adenocarcinoma; SARC, sarcoma; SKCM, skin cutaneous melanoma; STAD, stomach adenocarcinoma; TGCT, testicular germ cell tumours; THCA, thyroid carcinoma; THYM, thymoma; UCEC, uterine corpus endometrial carcinoma; UCS, uterine carcinosarcoma; UVM, uveal melanoma; N/A, not available.
