# Supplementary material for: Somatic mutations can induce a noninflamed tumour microenvironment via their original gene functions, despite deriving neoantigens
Source: Br J Cancer. 2023 Feb 2;128(6):1166–75. doi: 10.1038/s41416-023-02165-6 (PMC10006227; doi:10.1038/s41416-023-02165-6)
Supplement: Supplementary file 10 — Table S6 [file 41416_2023_2165_MOESM10_ESM.pdf]

Table S6. Correlation between driver or passenger mutation burden and immune activity scores.

Cancer type	Driver	*P* value	Passenger	*P* value
ACC	0.37	0.013	0.25	0.0035
BLCA	0.087	0.0086	0.078	0.12
BRCA	0.16	<0.0001	0.20	<0.0001
CA	0.34	<0.0001	0.37	<0.0001
CESC	0.23	0.0002	0.23	0.0002
CHOL	-0.22	0.21	-0.084	0.63
ESCA	-0.14	0.068	-0.11	0.13
GBM	-0.043	0.60	-0.041	0.61
HNSC	0.035	0.44	0.028	0.54
KICH	0.012	0.92	-0.029	0.82
KIRC	-0.0074	0.99	0.037	0.50
KIRP	-0.054	0.38	-0.006	0.33
LGG	-0.055	0.22	0.071	0.11
LIHC	0.060	0.26	0.032	0.56
LS	-0.013	0.78	0.047	0.32
LUAD	0.043	0.35	0.068	0.14
MESO	-0.17	0.13	-0.28	0.013
OV	0.12	0.084	-0.053	0.50
PAAD	-0.27	0.0002	-0.25	0.012
PCPG	0.016	0.84	-0.053	0.50
PRAD	-0.10	0.024	-0.045	0.33
SARC	-0.018	0.78	0.054	0.42
SKCM	-0.35	0.46	-0.19	0.69
STAD	0.15	0.0024	0.15	0.0027
TGCT	0.060	0.49	-0.13	0.14
THCA	-0.081	0.77	0.039	0.39
THYM	-0.39	<0.0001	-0.56	<0.0001
UCEC	0.13	0.0035	0.13	0.0049
UCS	0.32	0.017	0.42	0.0011
UVM	-0.24	0.037	-0.16	0.16

ACC, adrenocortical carcinoma; BLCA, bladder urothelial carcinoma; BRCA, breast invasive carcinoma; CA, colorectal adenocarcinoma; CESC, cervical squamous cell carcinoma; CHOL, cholangiocarcinoma; ESCA, esophageal adenocarcinoma; GBM, glioblastoma multiforme; HNSC, head and neck squamous cell carcinoma; KICH, kidney chromophobe; KIRC, kidney renal clear cell carcinoma; KIRP, kidney renal papillary cell carcinoma; LGG, brain low grade glioma; LIHC, liver hepatocellular carcinoma; LS, lung squamous cell carcinoma; LUAD, lung adenocarcinoma; MESO, mesothelioma; OV, ovarian serous cystadenocarcinoma; PAAD, pancreatic adenocarcinoma; PCPG, pheochromocytoma and paraganglioma; PRAD, prostate adenocarcinoma; SARC, sarcoma; SKCM, skin cutaneous melanoma; STAD, stomach adenocarcinoma; TGCT, testicular germ cell tumours; THCA, thyroid carcinoma; THYM, thymoma; UCEC, uterine corpus endometrial carcinoma; UCS, uterine carcinosarcoma; UVM, uveal melanoma.
