# Supplementary material for: Somatic mutations can induce a noninflamed tumour microenvironment via their original gene functions, despite deriving neoantigens
Source: Br J Cancer. 2023 Feb 2;128(6):1166–75. doi: 10.1038/s41416-023-02165-6 (PMC10006227; doi:10.1038/s41416-023-02165-6)
Supplement: Supplementary file 11 — Table S7 [file 41416_2023_2165_MOESM11_ESM.pdf]

Table S7. Correlation between neoantigen loads derived from driver/passenger mutations and immune activity scores.

Cancer type	Driver	*P* value	Passenger	*P* value
BLCA	0.055	0.28	0.062	0.22
BRCA	0.14	<0.0001	0.18	<0.0001
CA	0.35	<0.0001	0.39	<0.0001
CESC	0.22	0.0002	0.23	0.0002
GBM	-0.0086	0.91	-0.049	0.55
HNSC	0.10	0.027	0.13	0.0060
KICH	0.11	0.37	0.060	0.63
KIRC	-0.031	0.56	0.0045	0.41
KIRP	0.0022	0.97	-0.032	0.60
LIHC	-0.023	0.67	-0.060	0.27
LS	0.025	0.60	0.074	0.12
LUAD	0.0077	0.86	0.055	0.23
OV	0.13	0.064	0.024	0.74
PAAD	-0.15	0.058	-0.21	0.0062
PRAD	0.23	<0.0001	0.41	<0.0001
SKCM	-0.057	0.64	-0.0066	0.96
STAD	0.13	0.0092	0.15	0.0027
THCA	-0.070	0.13	-0.055	0.23
UCEC	0.12	0.012	0.11	0.012

BLCA, bladder urothelial carcinoma; BRCA, breast invasive carcinoma; CA, colorectal adenocarcinoma; CESC, cervical squamous cell carcinoma; GBM, glioblastoma multiforme; HNSC, head and neck squamous cell carcinoma; KICH, kidney chromophobe; KIRC, kidney renal clear cell carcinoma; KIRP, kidney renal papillary cell carcinoma; LGG, brain low grade glioma; LIHC, liver hepatocellular carcinoma; LS, lung squamous cell carcinoma; LUAD, lung adenocarcinoma; OV, ovarian serous cystadenocarcinoma; PAAD, pancreatic adenocarcinoma; PRAD, prostate adenocarcinoma; SKCM, skin cutaneous melanoma; STAD, stomach adenocarcinoma; TGCT, testicular germ cell tumours; THCA, thyroid carcinoma; UCEC, uterine corpus endometrial carcinoma.
